# Prevalence of musculoskeletal injuries and a proposal for neuromuscular training to prevent lower limb injuries in Brazilian Army soldiers: an observational study

**DOI:** 10.1186/s40779-018-0172-7

**Published:** 2018-07-27

**Authors:** Michele Zukauskas de Andrade Gomes, Carlos Eduardo Pinfildi

**Affiliations:** 1 Department of Human Movement Science, Federal University of São Paulo (UNIFESP), Campus Baixada Santista, Rua Silva Jardim, 136, Vila Mathias, Santos, SP 11015-020 Brazil; 2 Human Movement Science and Rehabilitation, Department of Human Movement Science, Federal University of São Paulo-Physical Therapy, Campus Baixada Santista, Rua Silva Jardim, 136, Vila Mathias, Santos, SP 11015-020 Brazil

**Keywords:** Physical therapy, Wounds and injuries, Primary prevention, Lower extremity, Proprioception, Military personnel

## Abstract

**Background:**

The activities carried out by soldiers in the army involve great physical demands and require intense trainings to perform combat-specific tasks. Musculoskeletal injury is a potential threat to the health and physical integrity of the soldier. This study aimed to evaluate the prevalence of lower limb musculoskeletal injuries among soldiers and to propose a training protocol to prevent the most frequent injuries.

**Methods:**

This observational (cross-sectional) study recruited a sample of 103 soldiers who required medical attention, from a total 202 new battalion soldiers. The medical records (paper and online) had a form of running text. All data collected were recorded by the registered physicians of the battalion medical post. The records were analyzed by the following variables: medical diagnosis, injury site, mechanism, type of treatment, time loss, existence of previous injury, and recurring injury.

**Results:**

A total of 112 musculoskeletal injuries were diagnosed in 71 soldiers, and other types of diseases/injuries were diagnosed in the other soldiers. Joint pain accounted for 55.4% of the diagnoses. The knee was the most affected site, while trauma and overload were the most common mechanisms of injury. Drug treatment was used most frequently, accounting for 58% of the cases. The majority of the sample obtained a temporary leave of absence for 1 to 6 days or not at all. Previous injuries and recurrence were not presented as risk factors for injury. With the data received, a protocol for the prevention of injuries to the lower limbs was proposed.

**Conclusion:**

This study indicated that the most frequent site of injury is the knee, and joint pain is the most common diagnosis. These results may support the necessity to develop a neuromuscular training protocol to prevent lower limb injuries, which we suggest to be applied in future studies.

## Background

The activities carried out by soldiers in the army demand great physical fitness and require intense training to perform combat-specific tasks. A soldier may experience musculoskeletal injuries because of the daily activities of running [1–6], marching [3, 4, 7, 8], specific exercises [3, 4, 9, 10], and sports activities within the unit [5, 8, 9, 11–13].

Musculoskeletal injuries are a potential threat to the health and physical integrity of the soldier [12] because the lower limbs are commonly affected by injuries, and soldiers complain of pain, most specifically in the knee joint [1, 3–5, 8, 9, 14–18], followed by the lower vertebral column [8–11, 14, 18], ankle [1, 3, 5, 8–11, 18], and leg [14, 18]. Several studies have shown that those injuries can occur in younger or less-trained individuals [1–3, 9, 15] and that previous injuries may be a recurrence factor [3, 4, 9, 16, 17]. Medical treatment and rehabilitation may generate high costs in addition to leaves of absence (time loss) [1, 4, 5, 8, 11, 12].

Soldiers in Brazil carry out daily military physical training (MPT), to acquire skills, speed, and agility, which are necessary for their profession. The MPT session consists of warm-up, the main work (running, basic strengthening, and/or sports and neuromuscular training), and finally, cooldown [19]. To assess the MPT performance, the military soldiers have to undertake the Physical Aptitude Test (PAT) [19] three times a year, which requires maximum effort to achieve the predicted index (values that soldiers need to reach based on Brazilian Military data). This test may cause injuries to new and untrained soldiers because of these high standards. The index varies according to age and sex as well as to pace and distance to be achieved. It ranges from insufficient to excellent. The PAT for men assesses running and military progression (PPM), including obstacle transposition, abdominal strength, push-ups, and bar exercises [19]. Like many other sports, the activities bring mechanisms of injury. However, there are differences in the specific activities [20], highlighting the importance of knowing the most common mechanisms of injuries in soldiers.

The studies conducted on injury prevention in the sports field are based on the particularity of each modality due to the different levels of exposure and mechanisms of injury. Studies related to preventive injuries in the military environment are limited because knowledge is needed for the military training, army routine, and the mechanisms of injury or simulations of the different activities. Furthermore, the difference in military training between each country, which is due to the action required by the Armed Forces, should be taken into consideration [4, 5, 8]. Therefore, consensus has been reached that preventive training should meet the needs of the soldier in a specific country.

Several studies in sports and military areas have demonstrated that exercises such as balance, running, agility, jumping, muscular strengthening, stretching, and corrective orientations can help to prevent injuries to the lower limbs [5, 21–27]. Considering the hypothesis that Brazilian soldiers commonly have lower limb injuries, incorporating neuromuscular training is necessary. In Brazil, no studies on the incidence or prevalence of musculoskeletal injuries in army personnel or preventative studies on these injuries were found. We hypothesize that Brazilian soldiers have a characteristic mechanism of injuries in the lower limbs due to the type of training. Thus, the objective of this study was to assess the prevalence of musculoskeletal injuries in the Brazilian army and to determine which injuries and mechanisms occurred most frequently in order to propose a neuromuscular training protocol to prevent these injuries.

## Methods

### Study design and sample details

This is an observational (cross-sectional) study. The study protocol was approved by the Ethics Committee for Research of the Federal University of São Paulo (UNIFESP) (approval number 326.888). This study was conducted in compliance with the Strengthening the Reporting of Observational Studies in Epidemiology (STROBE) Statement: guidelines for reporting observational studies [28].

A total of 202 soldiers aged between 18 and 19 years from a battalion of the Brazilian Army were recruited to participate in the study. In 2013, the battalion accepted 202 new soldiers (all men), called recruits. Among these 202 soldiers, 103 who required medical attention at the medical post were selected to participate in the study. Newly recruited soldiers were selected to study the prevalence and mechanisms of injuries because most studies have shown that injury incidence in recruits is greater than that in older soldiers who are more trained [1–3, 9, 15]. All these soldiers were of the same age and physical condition. The inclusion criteria were new soldiers who enrolled in 2013 and who had injury occurrence in the period between March and October. Musculoskeletal injury is physical musculoskeletal damage to the body [8] and requires the soldier to seek medical help. The exclusion criteria were soldiers who were from another military service on exchange, soldiers who had returned to training following a previous injury, and soldiers from other battalions.

The data were collected in November 2013 by a physiotherapist who had no access to any other information or medical records from the medical post. The medical records (paper and online data) had a form of running text. For this study’s design, all data collected were recorded only through what had been registered by the physicians. This explains why no demographic data were available in our study. A total of 103 medical record cards of the new soldiers were analyzed for the following variables: medical diagnosis, injury site, mechanism, type of treatment, time loss, existence of previous injury, and recurring injury.

The second phase of the collection took place on the same day through the enrichment of the paper medical record information, which was conducted via analysis of the online medical records. Subsequent to data collection, a survey of the main injuries and the mechanisms involved was carried out to propose an injury prevention training for the lower limbs.

Table 1 shows the variables “diagnosis,” “site,” and “mechanism” of the injury. All data collected per the inclusion criteria were named “components.” To make it possible to investigate the data using statistical models, the data were divided into five subgroups based on the diagnosis, the mechanism, and the site of injury. Concerning the variable “site,” the knee and ankle were considered separately, because compared to other injuries related to the lower limbs the literature presents that they have the highest incidence [1, 3–5, 8, 10, 14–18].

**Table 1:** Variables used to describe the subgroups of diagnosis and mechanisms of the injuries

Categories	Components
Diagnosis	
Joint pain	Pain, sprain, and joint pain
Muscular pain	Contracture
Inflammation	Tendinopathy, Hoffa’s fat pad inflammation, cistus, costochondritis, periostitis, dislocation, ligament injury, enthesopathy, and subdislocation
Wound	Cut, excoriation, trauma, and fracture
Degeneration	Chondropathy and patellofemoral pain syndrome
Injury site	
Spine	Cervical, dorsal, and lower vertebral column
Upper limbs	Head, chin, chest, fingers, hand, wrist, rib, elbow, and shoulder
Others (lower limbs)	Hip, leg, foot, heel, toes, triceps surae, tibia, and groin
Ankle	Ankle
Knee	Knee
Mechanism	
Running	Recreational run and military training running
Trauma	Fall, motorcycle accident, automobile accident, fall due to military training, and cut accident
Sports	Recreational soccer, push-up exercises using bars, fight, gym, and military training
Overload	Mechanical overload, biking, and field activities
No reason	Unknown mechanism

### Neuromuscular training proposal

Based on the collected data, a protocol was proposed for neuromuscular training to prevent injuries to the lower limbs, with emphasis on the needs observed during the training of soldiers in a battalion of the Brazilian army and on the mechanisms that most frequently caused injuries. A pre-training and intermediate assessment were suggested to better understand the consequences and possible improvements on the neuromuscular training, and a reassessment after 1 year is suggested.

### Pre- and postevaluations of soldiers

As suggested, an initial questionnaire was administered to determine the physical condition of the soldiers in addition to the noninstrumented assessments to measure the soldiers’ progress during the predicted year. The assessments should include jumping, balance, and agility in a circuit that would also serve as neuromuscular training, which could be measured by the time taken to complete the circuit.

### Statistical analysis

This study aimed to analyze the association among the variables studied. Fisher’s exact extension test was used to verify the association between injury sites, diagnosis, mechanisms, and time of medical leave. Our sample size was defined as “nonprobability sampling” because the soldiers were a convenience or availability sample of Brazilian army recruits. To test the null of independence of rows and columns in a contingency table with fixed marginal distribution, Fisher’s exact test was used. The descriptive level used was 5% with *P <* 0.05 as significant. For statistical analysis, the R package (version 3.0.1 2016) was used.

## Results

### Data distribution

After all data assessments, 71 soldiers were found to have 112 musculoskeletal injuries, and 32 soldiers had various complaints that were not characterized as musculoskeletal injuries. Table 2 shows the musculoskeletal injuries in absolute numbers and percentage, which demonstrates a significant association between diagnosis and injuries sites (*P <* 0.001).

**Table 2:** Numbers and percentage of diagnosis and injury sites[*n(%)*]

Diagnosis	Injury sites					Total
	Knee	Ankle	Spine	Upper Limbs	Other injuries in lower limbs	
Joint pain	19(17.0)	14(12.5)	14(12.5)	9(8.0)	6(5.4)	62(55.4)
Muscular pain	0(0.0)	0(0.0)	0(0.0)	1(0.9)	4(3.6)	5(4.5)
Inflammation	7(6.2)	0(0.0)	0(0.0)	4(3.6)	4(3.6)	15(13.4)
Wound	9(8.0)	0(0.0)	0(0.0)	11(9.8)	8(7.1)	28(24.9)
Degeneration	2(1.8)	0(0.0)	0(0.0)	0(0.0)	0(0.0)	2(1.8)
Total	37(33.0)	14(12.5)	14(12.5)	25(22.3)	22(19.7)	112(100.0)

Table 3 shows the results of the mechanism variable and injury site, and significant results were observed between them (*P <* 0.005). The most frequently observed mechanisms were trauma (26.7%) and overload activities (26.7%), followed by running and sports activities (18.8%).

**Table 3:** Mechanisms and injury site variables[*n(%)*]

Mechanism	Injury sites					Total
	Knee	Ankle	Spine	Upper Limbs	Other injuries in lower limbs	
Running	9(8.0)	8(7.1)	0(0.0)	0(0.0)	0(3.7)	21(18.0)
Trauma	10(8.9)	2(1.8)	2(1.8)	8(7.1)	8(7.1)	30(26.7)
No reason	3(2.7)	0(0.0)	3(2.7)	2(1.8)	2(1.8)	10(9.0)
Sports	3(2.7)	2(1.8)	3(2.7)	8(7.1)	5(4.5)	21(18.8)
Overload	12(10.7)	2(1.8)	6(5.3)	7(6.3)	3(2.6)	30(26.7)
Total	37(33.0)	14(12.5)	14(12.5)	25(22.3)	22(19.7)	112(100.0)

With regard to the treatments used on the soldiers, 58% were administered medication, 20.5% did not receive treatment, and 9.8% received two different types of treatment, physiotherapy (7.2%) and stitches/surgery (4.5%).

Table 4 shows the association between the time of medical leave and the injury site in soldiers stratified by medical leave of 1–6 days (35.7%), no leave of absence (33.9%), 7–15 days leave of absence (19.7%), and more than 15 days leave of absence (10.7%). The knee and ankle injuries resulted in the 7–15 days and 1–6 days leave of absence. The association between medical leave and injury sites showed significant results (*P <* 0.005).

**Table 4:** Data on medical leave and injury site variables[*n(%)*]

Medical leave	Injury sites					Total
	Knee	Ankle	Spine	Upper Limbs	Other injuries on lower limbs	
1–6 days	9(8.0)	9(8.0)	6(5.4)	9(8.0)	7(6.3)	40(35.7)
7–15 days	11(9.8)	1(0.9)	3(2.7)	3(2.7)	4(3.6)	22(19.7)
More than 15 days	10(8.9)	1(0.9)	0(0.0)	0(0.0)	1(0.9)	12(10.7)
No leave	7(6.3)	3(2.7)	5(4.4)	13(11.6)	10(8.9)	38(33.9)
Total	37(33.0)	14(12.5)	14(12.5)	25(22.3)	22(19.7)	112(100.0)

With regard to the risk factors of previous injury and recurring injury, the results showed that 67% of the sample did not have previous injuries. Prior to the study period, the injury was defined as any injury, regardless of the site injured, which occurred during the study period. Recurrence of the reported injury did not occur in 88.4% of the sample. These data were self-reported.

Fisher’s exact extension test [29] showed that there was an association between the variable injury site versus diagnosis (*P <* 0.001), mechanism (*P <* 0.005), and time loss (*P <* 0.003). No significant association was found between treatment and injury site (*p* > 0.115).

### Neuromuscular training circuit

**Fig. 1:**
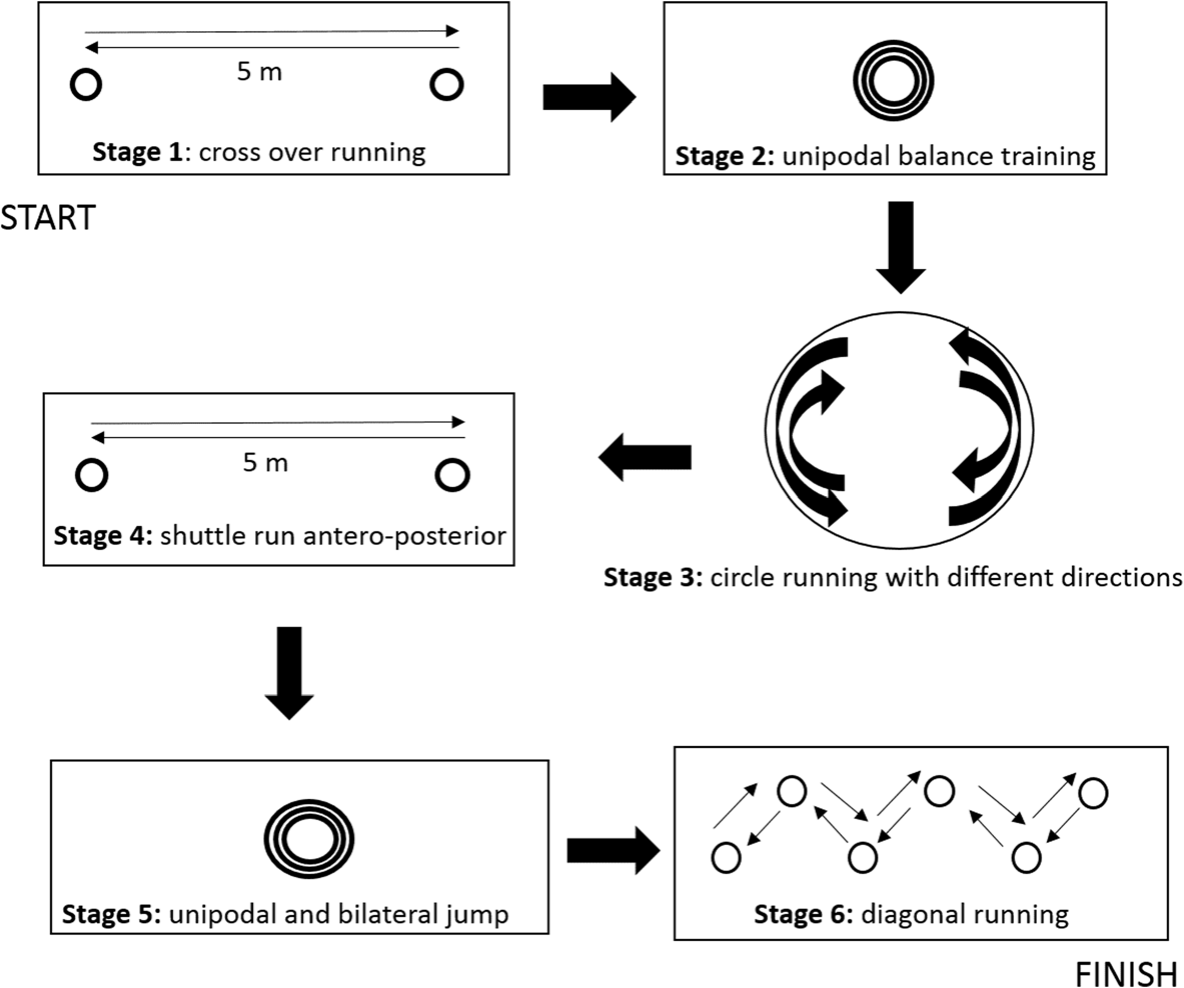
Neuromuscular training circuit with 6 exercise stations

The training session was suggested to be two laps of the proposed circuit (Fig. 1) with a 5–8 min rest interval. The training was a substitute for running, performed twice a week, since running is one of the main mechanisms of injury.

The description of the suggested neuromuscular training circuit is shown below:

- **Station 1:** Cross-over running [10, 24, 25], crossing the legs in lateral displacement; go and return over a 5 m course, with three repetitions, in the shortest time possible.
- **Station 2:** Balance [24–27, 30] on the trampoline, stand on one foot then bilateral for 30 s each; repeat twice.
- **Station 3:** Semicircular running [26, 31] involves changing direction at the verbal command of the therapist, for 1 min, with cones forming a semicircle.
- **Station 4:** Anterior and posterior running [24, 25, 32] over a distance of 5 m forwards and 5 m backwards, repeating 3 times in the shortest time possible.
- **Station 5:** Bilateral one-foot jump [10, 24–27, 30], repeated 10 times, on the trampoline, finishing with 10 two-foot jumps in each situation. The landing technique would be corrected for knee and foot alignment with the hips to reduce the impact forces and injury risk. In addition, this neuromuscular training improves the biomechanical quality of the movement [10, 33].
- **Station 6:** Running with 5 cones on the diagonal, a total distance of 5 m between the first and the last cone (zigzag) [10, 32], which is performed 3 times in the shortest time possible. On the first and second arrivals at the starting cone, the subject does balance training on the round proprioceptive disk for the right lower limb and 30 s for the left lower limb.

## Discussion

The present study is in agreement with previous studies on the incidence or prevalence of musculoskeletal injuries in soldiers, which also showed that the lower limbs are the most affected site of injury and complaint, especially the knee and ankle [1, 3–5, 8, 9, 14–18]. Concerning the injury site, Sell et al. [5] analyzed injuries of soldiers for a year and reported that the most frequent sites were the lower limbs, predominantly the ankle (18.2%) and the knee (13.1%). A Brazilian study by Neves et al. [34] on parachutists showed that the specificity of each group of soldiers is fundamental to understanding their injuries, and the authors observed that the foot and ankle are the most affected joints in parachutists. The present study showed that 33% of the injuries occurred to the knee, while the ankle was the fourth most affected site, together with the vertebral column (12.5%). The total injuries in the lower limb accounted for 65.2% of the sample. Considering that the soldiers were recently enrolled, many of them may not have practiced physical activities before enrolling, and furthermore, they are 18- to 19-year-old individuals without previous training focused on correcting biomechanical patterns with potential for injury, as pointed out by Sell et al. [5].

With regard to injury diagnoses, Hill et al. [16] showed that the most common injuries in soldiers’ knees were strain and sprain. Sell et al. [5] found that sprain (16.2%), mostly of the ankle, was the most frequent diagnosis. In New Zealand, Davidson et al. [1] studied the incidence of injuries in the lower limbs and diagnosed more sprains and strains in the ankle (35%) and knee (16%). In the present study, joint pain that involves sprain was the main diagnosis, accounting for 55.4% of the sample. The knee, ankle, and vertebral column were the most commonly diagnosed sites of injury. A possible decrease in these injuries could be achieved by the training proposed, which is based on agility and mechanical correction of the movements, aiming to prevent injuries.

Injuries from overload occurred with greater frequency than acute and traumatic injuries [7, 8, 18]. Davidson et al. [1] demonstrated that running and recreational sports were the most common mechanisms (28 and 25%, respectively), and 5 times more injuries were observed in recruits than in more trained individuals. In the present study, the most frequent mechanisms of injury were trauma and overload. Running was the second most frequent mechanism (18.8%), together with sports, which is different from the study of Sell et al. [5] who reported running as the main injury mechanism. Running mostly affects the knee and ankle. However, trauma and overload affect the knee more. These data led to the realization of decreasing the frequency of running, without damaging physical conditioning [10], to reduce probable overloads and/or traumatic events, as reported in the study by Dorotka et al. [17]. Rudzki [3] compared recruits who did the same physical training program, one group by running and the other marching with weights. The authors validated that the first group had more knee injuries.

In Finland, a study by Taanila et al. [8] reported that the most common mechanism of traumatic injury was in combat training or performing other physical exercises. In a British study by Wilkinson et al. [9], physical training, military training, and sports were the most common traumatic etiologies in soldiers. In the present study, field activities that correspond to the military trainings, such as overload activities, were differentiated. It was perceived that there are differences in mechanisms of injury among countries; for example, combat training was not so frequent in the Brazilian army. In Norway, Heir et al. [14] compared injury incidence in the three Armed Forces and reported that the lower limbs were the most affected site of injury, causing 2.1% of leaves of absence compared to 0.1% in the other two forces. In the present study, the injuries resulted in a time loss for a short period of time, 1 to 6 days, or did not result in leave of absence, as reported in some literature [4, 8]. The diagnoses with the longest time loss, over 15 days, were joint pain, inflammation, and degenerative pain, and the site was the knee.

This protocol was proposed because studies have shown the importance and capacity for injury prevention in the lower limbs and/or improvement in conditioning in sports and military activities [5, 21–27]. Exercises related to neuromuscular training should be reproducible and affordable to execute and may be carried out by soldiers during the MPT with the help of the physiotherapist and physical educators. The Finnish study by Parkkari et al. [22] on neuromuscular training performed by soldiers observed a decrease in the risk of ankle sprain, suggesting that this training should be routine. This training included exercises that do not characterize a specific sequence for the lower limbs; therefore, they were not chosen for the present study. The present study investigated the training according to the necessities and reality of Brazilian soldiers and the mechanisms of injury.

The study on injuries by Abt et al. [10] proposed running, agility, strengthening, and resistance exercises for soldiers, which resulted in improvement of their physical conditioning, and suggested further studies to identify injury prevention. Taanila et al. [4] emphasized that the unsatisfactory results in distance running and jumping performed by soldiers confirmed the predisposition to musculoskeletal injuries of the lower limbs, which shows that the training should involve these two modalities. Thus, the protocol includes exercises that involve less running and alternates with jumping, balance, and agility [5, 22, 24, 27], with the objective of improving sensorimotor training and minimizing injuries in the lower limbs.

Thus, the present study emphasizes that military physical training may consist of other exercise modalities, such as neuromuscular training that aims to prevent injuries and training characteristics inherent to the military profession. Brazilian training differs from that of other nations, for example, in addition to running, swimming, skiing, and bike training are also included [4, 8, 22]. Although the present study shows data not previously reported in the literature, our main limitation is that the data were collected from a single battalion. It is not possible to extrapolate our data and the proposal for neuromuscular training for all soldiers in any country, but after this study, we could anticipate the main mechanisms and injuries from Brazilian recruit soldiers, which provide us support to develop this proposal in further studies.

Future studies should include the proposed neuromuscular training to validate its efficiency in preventing injuries in the lower limbs of soldiers, as the literature can provide the basis and can prove its usefulness in the sporting environment. This training was presented to the battalion to analyze the possibility of adding it to the military physical training routine, not only for this battalion.

## Conclusion

The knee is the most frequently injured joint, and the most common diagnosis is joint pain. Trauma and overload are the most frequent mechanisms of injury, and the most commonly used treatment is medication. The time of medical leave due to injury was short in most cases, and the relationship with prior and recurring injury as relevant risk factors was not shown. The study promotes a neuromuscular training protocol, which is suggested to be practiced and improved in further studies.

## Abbreviations

MPT: Military Physical Training
PAT: Physical Aptitude Test
PPM: Military Progression
UNIFESP: Federal University of São Paulo

## References

[1] Davidson PL, Chalmers DJ, Wilson BD, McBride D (2008). Lower limb injuries in New Zealand defence force personnel: descriptive epidemiology. Aust N Z J Public Health.

[2] Rudzki SJ (1997). Injuries in Australian army recruits. Part I: decreased incidence and severity of injury seen with reduced running distance. Mil Med.

[3] Rudzki SJ (1997). Injuries in Australian army recruits. Part II: location and cause of injuries seen in recruits. Mil Med.

[4] Taanila H, Suni J, Pihlajamäki H, Mattila VM, Ohrankämmen O, Vuorinen P (2010). Aetiology and risk factors of musculoskeletal disorders in physically active conscripts: a follow-up study in the Finnish Defence forces. BMC Musculoskelet Disord.

[5] Sell TC, Abt JP, Crawford K, Lovalekar M, Nagai T, Deluzio JB (2010). Warrior model for human performance and injury prevention: eagle tactical athlete program (ETAP) part I. JSOM.

[6] Roy TC (2010). Diagnoses and mechanisms of musculoskeletal injuries in an infantry brigade combat team deployed to Afghanistan evaluated by the brigade physical therapist. Am J Prev Med.

[7] Knapik JJ, Reynolds KL, Harman E (2004). Soldier load carriage: historical, physiological, biomechanical, and medical aspects. Mil Med.

[8] Taanila H, Suni J, Pihlajamäki H, Mattila VM, Ohrankämmen O, Vuorinen P (2009). Musculoskeletal disorders in physically active conscripts: a one-year follow-up study in the Finnish Defence forces. BMC Musculoskelet Disord.

[9] Wilkinson DM, Blacker SD, Richmond VL, Horner FE, Rayson MP, Spiess A (2011). Injuries and injury risk factors among British army infantry soldiers during predeployment training. Inj Prev.

[10] Abt JP, Sell TC, Crawford K, Lovalekar M, Nagai T, Deluzio JB (2010). Warrior model for human performance and injury prevention: eagle tactical athlete program (ETAP) part II. JSOM..

[11] Yancosek KE, Roy T, Erickson M (2011). Rehabilitation programs for musculoskeletal injuries in military personnel. Mil Med.

[12] Nindl BC, Williams TJ, Deuster PA, Butler NL, Jones BH (2013). Strategies for optimizing military physical readiness and preventing musculoskeletal injuries in the 21st century. Mil Med.

[13] Jones BH, Canham-Chervak M, Canada S, Mitchener TA, Moore S (2010). Medical surveillance of injuries in the U.S. military descriptive epidemiology and recommendations for improvement. Am J Prev Med.

[14] Heir T, Glomsaker P (1996). Epidemiology of musculoskeletal injuries among Norwegian conscripts undergoing basic military training. Scand J Med Sci Sports.

[15] Ryu JH, Provencher MT (2011). Special considerations for ACL graft selection in the young, active military patient. J Knee Surg.

[16] Hill OT, Kay AB, Wahi MM, McKinnon CJ, Bulathsinhala L, Haley TF (2012). Rates of knee injury in the U.S. active duty army, 2000-2005. Mil Med.

[17] Dorotka R, Jimenez-Boj E, Kypta A, Kollar B (2003). The patellofemoral pain syndrome in recruits undergoing military training: a prospective 2-year follow-up study. Mil Med.

[18] Hauret KG, Jones BH, Bullock SH, Canham-Chervak M, Canada S (2010). Musculoskeletal injuries description of an under-recognized injury problem among military personnel. Am J Prev Med.

[19] Army News Brazil. O treinamento físico militar da força terrestre. http://www.eb.mil.br

[20] Heinrich KM, Spencer V, Fehl N, Poston WS (2012). Mission essential fitness: comparison of functional circuit training to traditional army physical training for active duty military. Mil Med.

[21] Brushøj C, Larsen K, Albrecht-Beste E, Nielsen MB, Løye F, Hölmich P (2008). Prevention of overuse injuries by a concurrent exercise program in subjects exposed to an increase in training load: a randomized controlled trial of 1020 army recruits. Am J Sports Med.

[22] Parkkari J, Taanila H, Suni J, Mattila VM, Ohrankämmen O, Vuorinen P (2011). Neuromuscular training with injury prevention counselling to decrease the risk of acute musculoskeletal injury in young men during military service: a population-based, randomised study. BMC Med.

[23] Coppack RJ, Etherington J, Wills AK (2011). The effects of exercise for the prevention of overuse anterior knee pain: a randomized controlled trial. Am J Sports Med.

[24] Olsen OE, Myklebust G, Engebretsen L, Holme I, Bahr R (2005). Exercises to prevent lower limb injuries in youth sports: cluster randomised controlled trial. BMJ.

[25] Lephart SM, Abt JP, Ferris CM, Sell TC, Nagai T, Myers JB (2005). Neuromuscular and biomechanical characteristic changes in high school athletes: a plyometric versus basic resistance program. Br J Sports Med.

[26] Longo UG, Loppini M, Berton A, Marinozzi A, Maffulli N, Denaro V (2012). The FIFA 11 + program is effective in preventing injuries in elite male basketball players. A cluster randomized controlled trial. Am J Sports Med.

[27] Emery CA, Meeuwisse WH (2010). The effectiveness of a neuromuscular prevention strategy to reduce injuries in youth soccer: a cluster-randomised controlled trial. Br J Sports Med.

[28] von Elm E, Altman DG, Egger M, Pocock SJ, Gøtzsche PC, Vandenbroucke JP, Initiative STROBE (2008). The strengthening the reporting of observational studies in epidemiology (STROBE) statement: guidelines for reporting observational studies. J Clin Epidemiol.

[29] Welsh AH (2011). Aspects of statistical inference.

[30] Van Beijsterveldt AMC, Van de Port IGL, Krist MR, Schmikli SL, Stubbe JH, Frederiks JE (2012). Effectiveness of an injury prevention programme for adult male amateur soccer players: a cluster-randomised controlled trial. Br J Sports Med.

[31] Whitehead PN, Schilling BK, Peterson DD, Weiss LW (2012). Possible new modalities for the navy physical readiness test. Mil Med.

[32] Kutlu M, Yapici H, Yonkalic O, Celik S (2012). Comparison of a new test for agility and skill in soccer with other agility tests. J Hum Kinetics.

[33] Willy RW, Davis IR (2011). The effect of a hip-strengthening program on mechanics during running and during a single-leg squat. J Orthop Sports Phys Ther.

[34] Neves EB, Souza MN, Almeida RMVR (2009). Military parachuting injuries in Brazil. Injury.

